# Traced to the Paste

**DOI:** 10.4269/ajtmh.18-0381

**Published:** 2018-08

**Authors:** Palak Patel, Amir Karimian, Jeffrey E. Sherwood

**Affiliations:** William Beaumont Army Medical Center, El Paso, Texas

**Question:** A 57-year-old male from southwest Texas with travel only to Taiwan 1 month prior develops new onset fever and abdominal pain. Stool microscopy is negative. Imaging and lesion aspirate is shown. What is the diagnosis?

**Answer:** Amebic liver abscess.

**Figure 1.**
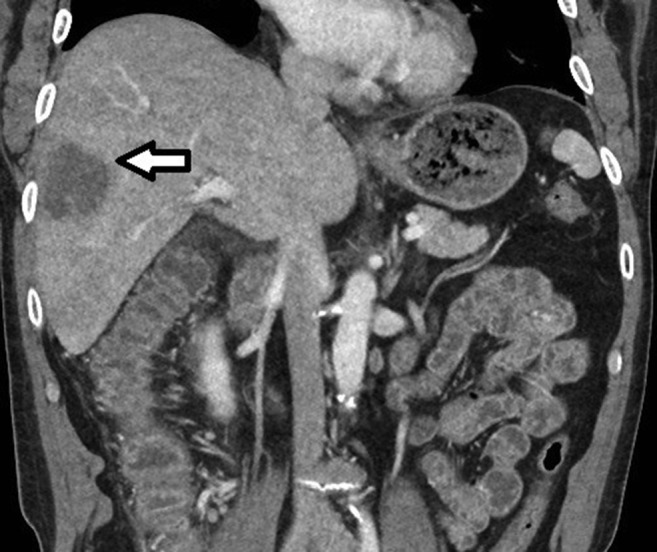
Computed tomography demonstrating amebic liver abscess in the right hepatic lobe.

**Figure 2.**
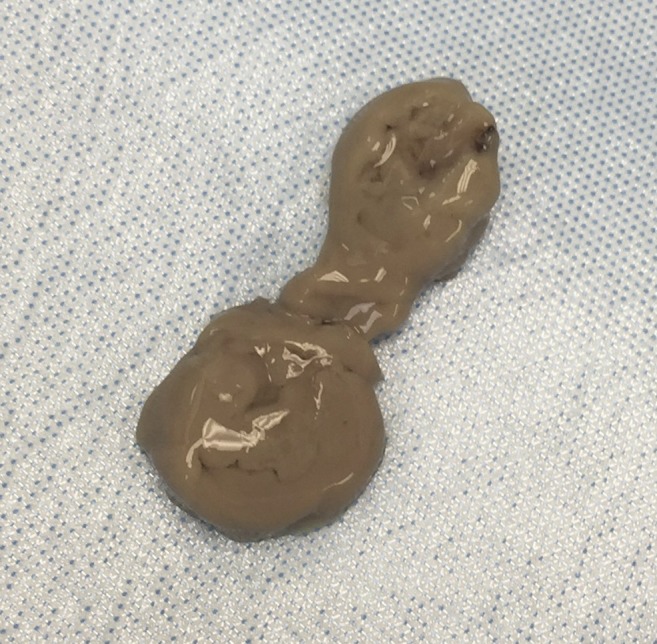
Aspirate of “anchovy paste” from the liver abscess. This figure appears in color at www.ajtmh.org.

A 57-year-old male originally from El Paso, Texas, with travel limited to Taiwan 1 month prior presented to our hospital with right upper quadrant abdominal pain, fever, and diarrhea. He admitted to consumption of night market food while in Taiwan. Laboratory evaluation revealed leukocytosis and elevated liver associated enzymes. Contrast enhanced computed tomography utilizing a multiphasic liver protocol revealed hepatic abscess in the right lobe measuring 3.6 × 4.7 × 4.1 cm (Figure 1). Intravenous ceftriaxone and metronidazole were initiated empirically. Because of persistent fever and concern for pyogenic liver abscess, aspiration was pursued. Classic “anchovy paste” material was discovered (Figure 2). Stool microscopy and baseline amebiasis antibodies were notably found to be negative. Stool and abscess fluid were subsequently tested utilizing the FilmArray^TM^ enteric polymerase chain reaction panel (BioFire Diagnostics, Salt Lake City, UT). Both samples were positive for *Entamoeba histolytica*. Routine abscess cultures were negative for bacterial coinfection. The patient completed courses of metronidazole and paramomycin with complete recovery. *Entamoeba histolytica* serology converted to positive and repeat imaging confirmed resolution of the abscess at follow-up.

Invasive amebiasis can be life threatening and early pathogen detection and treatment are of paramount importance. Traditional diagnostics such as light microscopy have low sensitivity and *E. histolytica* serology can specifically be falsely negative early in the course of the disease.^1,2^ Although not widely available, molecular techniques have quickly been recognized as the diagnostic standard and have been used to diagnose amebic liver abscess in several reports.^2,3^ This case highlights the pitfalls in utilizing conventional diagnostics as well as the utility of multiplex PCR to rapidly diagnose extraintestinal amebiasis.
